# Gig qualifications for the gig economy: micro-credentials and the ‘hungry mile’

**DOI:** 10.1007/s10734-021-00742-3

**Published:** 2021-08-03

**Authors:** Leesa Wheelahan, Gavin Moodie

**Affiliations:** Department of Leadership, Higher and Adult Education, Ontario Institute for Studies in Education, University of Toronto, 252 Bloor Street West, Toronto, ON M5S 1V6 Canada

**Keywords:** Micro-credentials, Competency-based education, Labour market precarity, Human capital theory, Skill-biased technological change, COVID-19

## Abstract

This paper argues that micro-credentials are gig credentials for the gig economy. Micro-credentials are short competency-based industry-aligned units of learning, while the gig economy comprises contingent work by individual ‘suppliers’. Both can be facilitated by (often the same) digital platforms, and both are underpinned by social relations of precariousness in the labour market and in society. They are mutually reinforcing and each has the potential to amplify the other. Rather than presenting new opportunities for social inclusion and access to education, they contribute to the privatisation of education by unbundling the curriculum and blurring the line between public and private provision in higher education. They accelerate the transfer of the costs of employment preparation, induction, and progression from governments and employers to individuals. Micro-credentials contribute to ‘disciplining’ higher education in two ways: first by building tighter links between higher education and workplace requirements (rather than whole occupations), and through ensuring universities are more ‘responsive’ to employer demands in a competitive market crowded with other types of providers. Instead of micro-credentials, progressive, democratic societies should seek to ensure that all members of society have access to a meaningful qualification that has value in the labour market and in society more broadly, and as a bridge to further education. This is a broader vision of education in which the purpose of education is to prepare individuals to live lives they have reason to value, and not just in the specifics required of particular jobs.

## Introduction

The ‘Hungry Mile’ refers to the line of men who walked along the wharves in search of work in Australia in the late 1920s and early 1930s during the years of Great Depression. Known as the ‘bull’ system, men would assemble in groups, hoping to be selected for a day’s work by the foreman. Stevens (2020) explains:

…foremen picked only the strongest and least ‘troublesome’ men to hire for the day. Scenes of chaos and desperation were common as foremen casually tossed ‘job tickets’ into the air to be snatched up by the quickest men. Those who succeeded in gaining a day’s work were then exposed to dangerous and backbreaking working conditions.

The gig economy takes a different form today, but it is still underpinned by the same ruthless logic and brutality as the bull system. The gig economy includes contingent work underpinned by digital platforms which mediate between individual ‘suppliers’ of services, goods, or other forms of labour and customers (Rani et al., 2021). It also includes other forms of contingent work such as freelancing, consulting, labour hire, and other forms of casual work which are not necessarily mediated through digital platforms (World Economic Forum [WEF], 2020). Micro-credentials are the quintessential expression of human capital theory in higher education, and their focus, form, and structure have significant potential to support the gig economy. While definitions of micro-credentials are not fully settled, there is an emerging consensus that they are short courses aligned with industry which are substantive ‘enough’ to be counted towards a full qualification (Kato et al., 2020). In turn, micro-credentials may consist of smaller units of learning, such as digital badges or digital certificates.

The purpose of this article is to outline the social and policy contexts in which micro-credentials have emerged, explain their potential to underpin the gig economy, and to problematise the taken for granted assumptions in policy that valorise them. Like many other policy incursions into higher education based on human capital theory, micro-credentials are presented as an antidote to problems of elitism in higher education. Opponents of micro-credentials are cast as those who wish to maintain higher education as an ivory tower and support elite structures of higher education, who are conservatives resistant to change and who deny any role for higher education in supporting people to gain credentials they need for a meaningful career. In contrast, proponents of micro-credentials argue that they are putatively promoting social inclusion, self-realisation, personalisation, and student-centred learning, while serving employers interests more closely (Wills & Xie, 2016). They are argued to have the potential to democratise access to higher education by lowering the cost of its acquisition (Willis III et al., 2016, p.31). They will revolutionise higher education, for the better (Cirlan & Loukkola, 2020).

We argue that micro-credentials are gig credentials for the gig economy. Their potential to underpin contingent, precarious work is greatest for those who are the most disadvantaged. Micro-credentials do little to challenge the hierarchical structuring of higher education, other than add an additional income stream for universities, including the most elite universities. Those without the access to elite occupations provided by the elite universities must take on more risk to ‘second-guess’ the requirements of the labour market so that they have the ‘right’ skills needed at the right time for the right job. Micro-credentials reflect the outsourcing and cost-shifting of employers’ internal professional development training to individuals who must demonstrate that they are ‘market ready’ without needing much—or any—on-the-job training (Brown & Souto-Otero, 2018). They represent the deeper incursion of the discourse of employability and competency-based education into higher education. The biggest problem is that they feed the myth perpetuated by human capital theory that the right ‘credential’ (or qualification) at the right time will enable individuals to ‘break through’ social congestion in the labour market and get a job aligned with their qualification at a level of skill commensurate with the level of the qualification (Brown et al., 2020; Livingstone, 2019), or, maybe, just a foot in the door to the labour market.

There are three main sections in this article. The first section provides the context for the emergence of micro-credentials and the niche they are meant to fill. It demonstrates how micro-credentials are congruent with human capital theory and its closely related cousin, skill-biased technological change (Brown et al., 2020). This provides the setting for the second section, which illustrates the link between micro-credentials, precarious employment, and the gig economy. The third section considers problems raised by micro-credentials: they seek to change supply of qualifications or credentials to solve a problem of demand for labour; they reorient higher education from educational purposes to employment purposes; they seek to divert students from substantial credentials with substantial value to micro-credentials with micro value; they further seek to discipline universities; and they extend the privatisation and marketisation of higher education.

The article would be too long to develop a social democratic alternative to the problems raised by late capitalism that micro-credentials were developed to address, but in conclusion, the paper refers to the authors’ argument that all members of society should have access to post-secondary qualifications which serve three roles though with different emphases and orientations: to prepare graduates for work, to develop graduates’ capacity to understand the world and to participate in social and political life, and to undertake further education (see Moodie et al., 2019; Wheelahan & Moodie, 2017). This is a broader and more inclusive vision of education in which the purpose of education is to prepare individuals to live lives they have reason to value (Nussbaum, 2000; Sen, 1999) and not just in the specifics required of particular jobs.

## Dominant rationale for higher education: human capital

Human capital theory argues that education increases peoples’ skills which increases their productivity in work which in turn increases economic output (Schultz, 1961). There is a direct linear connection between these elements at the level of the individual, group, enterprise, industry sector, and nation (Becker, 1962, 1964). Learning equals earning. That is, the direct relation between increasing investment in education and increasing economic output posited by human capital theory is said to increase graduates’ incomes, firms’ revenues, sectors’ economic contributions, and nations’ gross domestic products. A person’s position in society and in the labour market is a result of their investment in education, and is fundamentally meritocratic. This orthodoxy has underpinned education policy for more than four decades in which the purpose of education is to meet the needs of the labour market and the economy. In broad terms, the sum of qualifications or measures of educational attainment are taken as a rough proxy for a country’s stock of human capital (Centre for Educational Research and Innovation, 1998, p.16). The OECD (2020) produces *Education at a Glance* each year, which reports on the ‘stock’ of member countries’ human capital as measured by years of education and levels of attainment. As part of their broader critique of this approach, Brown and Souto-Otero (2018, p.2) explain that qualifications are posited by human capital theory as the mechanism used by individuals to ‘signal the potential of certain attributes that are difficult to observe at the time of recruitment but are viewed as relevant for an individual’s productive capacity’ (see also Bills, 2016).

Initially, human capital theory was descriptive: it was used to explain economic gains from expanding education; and it was also used to explain why individuals, companies, and governments increased their spending on education. Human capital theory then also became normative: advocates argued that investment in human capital should be increased to increase economic rewards. Since the 2000s, human capital theory has become increasingly prescriptive, with advocates arguing that postsecondary education should be increasingly concentrated on and then restricted to programs thought to have most economic benefit. As we argue more fully later, much of the advocacy for micro-credentials is a prescriptive form of human capital theory, or more precisely, its variant skill-biased technological change.

Human capital theory was consistent with increasing average rates of return for longer periods or higher levels of education until around the 1970s. Social mobility through education was possible after the Second World War as a consequence of the growth of white collar jobs in private and public sector bureaucracies in the 1960s (Brown et al., 2020, p.122), but recent decades have been characterised by stagnating and declining rates of employment, pay, and conditions under the new conditions of finance or market capitalism (Livingstone, 2019). From about 2010, the average premium for education started to flatten; employment rates fell, though they remained well above employment rates of those with less education; rates of underemployment have increased (Brown et al., 2020); and there are increasing mismatches between the field in which graduates are educated and the field in which they are employed (World Economic Forum and Boston Consulting Group, 2015).

A persistent problem for human capital theory is that the relation between years or level of education and economic gain observed on average belies marked disparities of rates of return by age, gender, ethnicity, geography, field, and other factors (Lauder et al., 2018). That is, the same investments in education do not result in the same kinds of outcomes for all. This is a rationale for human capital theory’s close relation and skills-biased technological change.

### The family squabble between human capital and skill-biased technological change

Brown et al. (2020) compare and contrast orthodox human capital theory and skill-biased technological change (SBTC) theory. Orthodox human capital theory posits that more education leads to more skills which leads to higher productivity and from there to a more productive nation with a higher gross domestic product (Becker, 1964). The supply of educated labour elicits demand from the labour market and leads to higher wages as employers respond to the higher productivity of more highly educated individuals (as explained by Brown et al., 2020, p.124 in their critique). In contrast to human capital theory which posits that education drives demand for skill, skill-biased technological change (SBTC) theory posits (broadly) that technological change elicits demand for skilled (educated) labour, so the arrow of causation between education and technology is reversed (Goldin & Katz, 2010; Machin, 2004). It also takes field or subject into account, which accounts for higher returns for some areas than others. SBTC focuses on the ‘right kind’ of skills that are needed for higher productivity and as a way of explaining the increasing inequality between high and low skilled workers.

In a very influential report, the World Economic Forum and Boston Consulting Group (2015, p.1) argue that the problem is that education has failed to teach the ‘right’ skills defined first as employability skills, and now as twenty-first century skills which include ‘critical thinking, problem-solving, persistence, collaboration and curiosity’. According to this approach, using years of educational attainment as the measure of human capital is too blunt because the quality of education varies within and between countries, and measures such as the OECD’s Program for International Student Assessment (PISA) and Programme for the International Assessment of Adult Competencies (PIAAC) provide better insights because all are measured in the same way (Gift & Wibbels, 2014). An extension of this argument about the primacy of skills is that for individuals, the right signals are not credentials or qualifications which indicate educational attainment, but specific employment skills which can be demonstrated through holding the ‘right’ micro-credentials.

## Micro-credentials to the rescue

Micro-credentials are part of a group of ‘alternative credentials’ (Kato et al., 2020), and while they are defined in different ways, in most cases they are substantial ‘enough’ to count towards a full qualification. Kato et al. (2020) report that many jurisdictions have micro-credentials of about six months or similar duration. Micro-credentials may be made up of smaller components such as digital badges and micro-courses and micro-certifications. No doubt, the definition of micro-credentials will cohere over time as jurisdictions borrow policy by incorporating them in their qualifications’ policies and frameworks.

Micro-credentials are mostly self-paced and associated with online learning, but they can also be offered face-to-face, in hybrid or blended modes which include face-to-face, group, and online components (Kato et al., 2020). Their development has been facilitated by the development of digital platforms that can safely record, store, and transmit these micro-units of learning between institutions (Keevy & Chakroun, 2019). The first use of these technologies on a mass scale was for MOOCs (massive online open courses), and these platforms have developed since then so that they can capture ‘all’ learning, including units of micro-learning such as badges. Keevy (2018, p.26) says that ‘digital technology is also expected to offer new credentialing methods and systems that can capture, recognise and validate a broader range of learning outcomes in the era of lifelong learning’.

Micro-credentials are focused explicitly on the labour market. For example, the New Zealand Qualifications Authority (n.d.) explains that:

A micro-credential certifies achievement of a coherent set of skills and knowledge; and is specified by a statement of purpose, learning outcomes, and strong evidence of need by industry, employers, iwi^1^ and/or the community.

Think tanks and philanthropic trusts are promoting micro-credentials and are helping to drive institutional change by embedding micro-credentials within different jurisdictions (Greene, 2019). There is a great deal of policy attention on micro-credentials by governments and international government organisations. For example, the Australian government is moving towards incorporating micro-credentials as a permanent part of the Australian Qualifications Framework (Department of Education Skills and Employment, 2020), while the Government of Aotearoa New Zealand has already done so (New Zealand Qualifications Authority n.d.). The Canadian provincial Government of Ontario (2020) is supporting the widespread implementation of micro-credentials, while several state governments in the USA are using micro-credentials in their teacher development programs, and exploring how they can be incorporated in evaluation of teachers (Berry & Byrd, 2019). The OECD (Kato et al., 2020), UNESCO (Chakroun & Keevy, 2018), and European Commission are working on micro-credentials, with the European Union establishing a Common Microcredential Framework to enable the recognition and portability of micro-credentials (Konings, 2019). UNESCO has a project to develop ‘World Reference Levels’ to enable the recognition of all types of credentials, including micro-credentials, digital badges, and other forms of micro-learning and is exploring how this can be automated through digital platforms accessed by individuals, employers, educational institutions, governments, and other interested parties (Chakroun & Keevy, 2018).^2^ Work is proceeding in Continental Africa to achieve similar results (JET Education Services, 2020).

This policy attention on micro-credentials has intensified during the COVID-19 pandemic which has accelerated their development as governments have sought to respond to massive increases in unemployment as a consequence of quarantining measures, and universities (at least in wealthy countries) have sought new markets to replace enrolments by international students who cannot leave their country of origin. For example, the Australian government introduced and funded a new under-graduate ‘higher education certificate’ of six-months duration as an explicit response to the COVID-19 pandemic (Commonwealth of Australia, 2020). The provincial Government of Ontario in Canada introduced considerable funding for micro-credentials, also as an explicit response to COVID-19 (Government of Ontario, 2020).

Proselytisers for micro-credentials portray them as tools of democratisation where curriculum is freed from restraints associated with universities, and accessed through private-for-profit entities. For example, Willis III et al. say: ‘With the proliferation of digitally-connected learning tools, institutional control of educational curricula has been democratized with free or low-cost tools like YouTube, Khan Academy, MOOCs, and many others’ (Willis III et al., 2016, p.31).

A key argument for micro-credentials is that the fast pace of change in work means that short, sharp episodes of learning just in time and just for now are needed to keep pace. Individuals, it is argued, shouldn’t have to go back to do a new credential when all they need to do is to reskill in particular areas (Oliver, 2019). And such learning needs to be more tightly integrated into ‘what industry wants’. For example, Oliver (2019, p.13) says that ‘Micro-credentials are a key opportunity for providers to achieve better integration with employers’. This takes for granted that this is what universities *should* be doing. Digital Promise is a US organisation signed into law by President Bush in 2008, launched by President Obama in 2011, and funded by the US Department of Education and various large philanthropic trusts. It explains that: ‘Micro-credentials provide a pathway to personalizing and recognizing professional learning. They allow employers to verify the skills their employees demonstrate, regardless of where and how they learned them’ (Digital Promise, 2020).

A further argument for micro-credentials is that they free individuals to choose what they will learn, how, when, and in what ways. They will putatively provide disadvantaged students with access to cheaper learning as they build towards a full credential and this can contribute to widening access to hitherto excluded groups. Micro-credentials can respond to the changing needs of the workplace by allowing individuals to upskill when and how they need to, and (it is claimed) probably for lower cost. Indeed, the language of empowerment, self-regulated learning and learner autonomy, personalisation, intrinsic self-motivation, and self-realisation are associated with micro-credentials in the same way as they were for MOOCs (see Wills & Xie, 2016).

Micro-credentials are generally based on competencies (Digital Promise, 2020) and consequently are also an incursion of this model of curriculum into higher education from vocational education where it originated and where it dominates. The language of learning outcomes, employability skills, graduate attributes, and now twenty-first century skills has dominated higher education for some time as part of the requirement for universities to be responsive to employers’ needs and students’ vocational aspirations (Walker, 2016). However, micro-credentials are more explicitly based on ‘tighter’ models of competencies which are linked to skills required at work. Deng (2020) explains that competency-based models of curriculum are derived from the field of human resource management, and are managerial concepts rather than curricular concepts. The explicit purpose of competency-based curriculum is to tie education directly to workplace requirements and roles. Micro-credentials are a vehicle through which this is achieved.

SBTC theory is helping to drive the development of and legitimation of micro-credentials based on the putative deficits of education in failing to provide individuals with the specific knowledge and skills needed by the labour market, and because of its suspicion of the value of qualifications. There is a direct line between higher technological skills and micro-credentials which have been produced to help meet demand for those skills. Micro-credentials can produce ‘in-demand’ skills at the right time, result in more effective skills assessment and recognition, and ‘fill the gap between academic programmes and the skills required by the labour market’ (Cirlan & Loukkola, 2020, p.15). Lauder et al. (2018, p.500) explain that for proponents of SBTC, ‘the notion of skill appears closer to an account of the relationship between education and the economy than that of the credential’. They explain that the ‘new databases have enabled SBTC theorists to operationalize elements of the unobserved skills that they have been seeking’ (Lauder et al., 2018, p.500).

### Investing in human capital as a safety net

As work has become more precarious, individuals must shoulder greater responsibility for risk in the labour market. This is particularly the case for those in external labour markets, where their occupational progression is not as clear as it is for those in regulated professions (such as nursing or medicine), or as in internal labour markets characterised by internal progression within firms (Yu, 2015). Those in external labour markets must ‘second guess’ the requirements of the labour market and build a skills profile that ensures that they can ‘hit the ground running’ in commencing new jobs (Brown & Souto-Otero, 2018). Micro-credentials are one way they can do this. For example, Burt and Gormley (2021) explain that the ‘value’ of alternative credentials such as micro-credentials is that employers need ‘to fill roles more quickly [and] are unable to wait for certain skills to evolve their way into the workforce. Job seekers need to be ready to quickly learn and document their proficiency with new skills and abilities to remain relevant and essential.’

The diminution of the welfare safety net over the last four decades of neoliberalism (Harvey, 2007) means that education is the principal safety net, and individuals must make sound investments, and if they don’t, it is because of the decisions they have made. Brown et al. (2020, p.35) explain that neoliberalism sought to increase individual responsibility for the creation of human capital as a way of encouraging ‘market incentives for individual enterprise’. They explain that the sole means by which individuals can improve their life chances is by investing in their human capital, as the basis of their participation in the labour market. The state no longer provides broader social insurance and welfare programs. Brown et al. (2020, p.39) explain: ‘This is human capital in the raw; without it, the chances of thriving in a neoliberal society are remote. Students and workers are to stand naked in the market, save for their credentials.’

On-demand micro-credentials reflect the fragmentation of occupations and increase precarity of the labour force. They reflect the skills discourse, and the need to have the ‘right’ skills at the right time. The cost is often borne by individuals seeking to break into or upgrade their position in the labour market, or prove that they are indispensable. Micro-credentials reflect employers’ outsourcing of internal training and development to individuals. For example, non-formal work-related learning in Australia fell by 12.4 percentage points from 2005 to 2016/17 (Australian Bureau of Statistics, 2017). Training intensity also declined, from a median of 28 hours in 2007 to 24 hours in 2017 (OECD, 2019a, p.19). Livingstone (2019) found similar declines in non-formal work-related learning in his study of Canada. Employers cut their investment in their employees’ training by around 40% over the last two decades in Canada (Hall & Cotsman, 2015), the UK (Green et al., 2013), and the USA (Cleary & Van Noy, 2014, p.1)

### The gig economy: micro-credentials as a response to precariousness in the labour market

The links between micro-credentials and the gig economy are still emerging, but are taking more definite shape. Just as micro-credentials are broader than those mediated by learning platforms, the gig economy doesn’t refer just to Uber or food delivery apps although these are possibly the most visible and their workers seem to work in the most difficult and precarious conditions. The gig economy refers not only to platform work, but also to other forms of contingent work that include freelancing, consulting, labour hire, and other forms of casual work (WEF 2020). The gig economy is characterised by precariousness in employment, and lack of access to pay and conditions associated with regulated jobs in the formal economy. The International Labour Organization (ILO) (2019, p.6) reports that 61% of the world’s workforce was in informal employment in 2016 who, by and large, also do not enjoy any social protection. The informal economy is, of course, a much bigger phenomenon in low-income countries, but its importance is rising in high-income countries as well. There are always problems with and debates about definitions of the informal economy, so the notion of precarious work is perhaps more helpful. For example, in discussing Australia, Stanford and Pennington (2019) explain that the resurgence in precarious employment takes many forms, including independent contracting, casual and temporary work, and on demand positions. In 2017, the percentage of Australians in the labour force who were in full-time paid work with leave entitlements fell to just under 50%, for the first time (Carney & Standford, 2018, p.17).

Stanford and Pennington (2019, p. 22) refer to the gig economy more broadly as the expansion of the on-demand economy and insecure work, which are the result of the conjunction of macroeconomic, regulatory, and political developments (p.22). The changes in the economy and in society are the result of the triumph of financial capitalism, the pursuit of shareholder profit, and the decline of the welfare state (Standing, 2009; Therborn, 2012). Work has become more precarious as power has shifted from labour and unions, and as the welfare safety net has been largely removed. Bourdieu (2001) argues that precarity has come to be the doxa governing late capitalism. While different segments of society and of the working class are affected in different ways by precariousness (Standing, 2011), nonetheless all live in its shadow, including those in more traditional ‘secure’ forms of work. Bourdieu (2001, p. 29) explains we now have ‘*institutionalized precariousness*’ and that:

Thus has come into being an economic regime that is inseparable from a political regime, a mode of production that entails a mode of domination based on the *institution of insecurity*, domination through precariousness: a deregulated financial market fosters a deregulated labour market and there the casualization of labor that cows workers into submission.

In Australia, there is a term that refers to the casual cruelty that results from this domination through precariousness—the ‘spill and fill’. Wyborn and Vautin (2013) explain, in rather neutral terms, that: ‘A spill and fill is a restructuring process whereby a range of positions in a workplace are made redundant and the employees filling those positions must reapply for the smaller number of newly created positions.’ And these new positions may be at lower levels with lower pay.

The growth of insecure work is not a consequence of technology, but technology does facilitate how it is managed and supervised (Stanford & Pennington, 2019). According to the World Economic Forum (2020), platform work and services are still a relatively small but fast growing segment of the gig economy, facilitated in part by platforms. Stanford and Pennington (2019, p.14—emphasis in original) explain that ‘***the growing precarity of work, including in digitally mediated on demand jobs reflects the evolution of social relationships and power balances, more than technological innovation in its own right****’.*

Platform learning has also facilitated the massive expansion of micro-credentials because of its capacity to digitise, store, and share outcomes of small components of learning. But this is far from a neutral technology. Rather, it is an educational response to increasing precarity in the labour market. As Means (2018, p.326) explains: ‘Platform learning harnesses the operating capabilities and logics of digital platforms such as Uber and Amazon to imagine synergies between on-demand labor and on-demand learning, transforming living into learning, and learning into labor’. He explains that these learning platforms ‘imagine learning and work futures [which are] reflective of the commercial and technocratic values and rationalities immanent to Silicon Valley and corporate technology more broadly’ (Means, 2018, p.327).

However, the notion of platform learning goes beyond the specific programs offered by the apps and the gig jobs that they are tied to. Rather, they reflect a shift in the relation between education and work which has emerged as a consequence of the increasing precarity of work in late capitalism. Means (2018, p327) explains that ‘the concept of *platform learning* as a speculative discourse… internalizes emergent conceptions of *education and work* within the on-demand economy of late capitalism’. Rani et al. (2021, p.23) explain that: ‘Platforms are redefining the relationship between formal education and access to work, as worker profiles, ratings and reputation are vital for accessing work’. And micro-credentials are one element of building a reputation and demonstrating possession of specific skills.

As an illustration, the OECD (2019b, p. 111) explains that: ‘Several large online platforms that facilitate the hiring of freelancers also offer online skills tests, where freelancers can take multiple-choice quizzes on various skills, across a variety of skills domains’. Seek Limited (n.d.), which is a private for profit company listed on the Australian Stock Exchange, started as ‘an online version of print employment classifieds’ and soon developed SEEK Learning (2019), ‘connecting job seekers with relevant, nationally recognised courses’. Platform-based work is not far from the ‘Bull’ system where foremen had discretion over who to choose to work for a day and to toss job tickets out to see who caught them. Micro-credentials and the gig economy are mutually reinforcing and will grow in importance, with both facilitated by platforms—sometimes the same platform which links the two. For example, Rani et al. (2021, p.185) report that about ‘20 per cent of respondents on freelance platforms reported that they had completed classes or training’ to improve their skills and/or enhance their profiles and obtain certification that they had done so. They are also mutually reinforcing because both are underpinned by social relations of precariousness.

## Problematics of micro-credentials

Micro-credentials raise several problems: they seek to change the supply of educational qualifications or credentials to solve a problem of demand for labour; they reorient higher education from educational purposes to employment purposes; they seek to divert students from substantial credentials with substantial value to micro-credentials with micro value; they further seek to discipline universities; and they extend the privatisation and marketisation of higher education.

### Change education supply to solve a problem of demand

Micro-credentials are presented as a key way in which the ‘skills problem’ and skills mismatches can be addressed. But as Brown et al. (2020, p.133) explain the problem is not skills, it is the availability of jobs: ‘The fundamental problem is not that there is a shortage of the relevant skills that employers demand but that there is a lack of good-quality jobs. The problem that needs to be addressed is not labor scarcity but job scarcity.’ Over time, there has been a shift from standard to non-standard forms of employment, and a decline of jobs in the middle of the skills spectrum (OECD, 2015) leading to congestion in labour market (Livingstone, 2019). Brown et al. (2020, p.124) say that there is a danger of:

defining economies and societies by dominant or emergent technologies or level of scientific knowledge, whatever label is applied: “industrial,” “information,” “knowledge,” or “digital” economy. Although capitalism can change its spots, it’s still capitalism…The education system cannot compensate for market capitalism.

In a congested labour market and expanding higher education, qualifications are a necessary defensive tool in the labour market rather than a differentiating factor for the majority of jobs (Bills, 2016; Brown & Souto-Otero, 2018, p.2). Individuals must differentiate themselves further. Micro-credentials are one way they can do this. This is not realisation of the self as presented by progressive discourses which promise social inclusion, access, diversity, and democratisation. Rather, it is the modern way in which individuals participate in, and compete with each other in, the hungry mile.

Micro-credentials have the potential to contribute to the fragmentation of occupations as employers are in a position to stipulate that applicants must have particular sets of skills related to particular tasks, rather than whole qualifications that underpin occupations. This has certainly been the experience in vocational education, where versions of micro-credentials have existed for some time (as with ‘skill sets’ in Australia) (Wheelahan, 2016).

### Reorient education

Human capital theory reorients the ends of education from the development of knowledge to the development of productive workers. Skill-biased technological change reorients the curriculum of education from knowledge to skills, and from educational skills to job skills. Micro-credentials greatly amplify both changes. They also greatly atomise and fragment skills development. Not only are micro-credentials much smaller than standard credentials, they also can be taken in any order and in any combination, or alone as self-contained credentials. This makes all but impossible the development of sustained knowledge and skills, notions of sequence or hierarchy, and thus of deep knowledge and skills (see Wheelahan & Moodie, 2021).

Micro-credentials further shape higher education as an instrument of microeconomic change to serve the economy and a market society (Buckner, 2017). They achieve two goals simultaneously: first, they help to discipline the curriculum in higher education so that it is more explicitly focused on work (Muller & Young, 2014); and second, they help to discipline higher education institutions so that they are more market oriented and responsive (Marginson, 2006). For example, the Australian Government Minister for Education, Dan Tehan (2020a) explained in a speech that: ‘The development of microcredentials will drive innovation and provide an additional income stream for universities, while making them more efficient, relevant to industry and responsive to the requirements of domestic students.’ Based on the old adage, ‘never let a good crisis go to waste’, he says: ‘Our Government wants short courses to be a permanent fixture of the Australian education system’ (Tehan, 2020b). It is clear that the Australian government is funding micro-credentials during the pandemic to drive more long-lasting change, which will embed them as a key component of higher education, including in the Australian Qualifications Framework (Department of Education Skills and Employment, 2020).

### Divert students from substantial credentials with substantial value to micro-credentials with micro-value

Unlike substantial educational credentials, micro-credentials do not seek to develop graduates’ capacity to understand the world and to participate in social and political life. Their sole aim is to improve employment outcomes. They are new and do not have a widely shared definition. There is therefore not strong statistical evidence of micro-credentials’ employment outcomes, but the limited evidence currently available suggests that micro-credentials do not have strong employment outcomes and that they are certainly lower than for substantial credentials.

Bailey and Belfield (2017) analyse USA national, survey, and college-system-level datasets to conclude that stackable credentials which include certificates of up to a year’s duration ‘show only weakly positive and inconsistent gains from these award combinations’. Burns and Bentz (2020, p. 3) analyse data from the USA 2012/14 Beginning Postsecondary Students Longitudinal Study to find students’ employment outcomes three years after ending their study. Some 72% of students who completed a certificate of up to a year’s duration were employed, which is markedly higher than 59% of students who did not complete a certificate. However, of those who were employed, there was no statistically significant difference in median salary between certificate completers and non-completers.

Ositelu (2021, p. 13) report the results of regional focus groups of 48 adults from Atlanta, GA, and Richmond, VA, to find that ‘Many participants in the focus groups believe that their program helped them to earn the certificate they needed to get a job and improve their skills’. However, it appears that their hopes don’t match the reality of the outcomes they achieve. Ositelu (2021, pp. 10-11) analysed data from the USA National Center for Education Statistics’ 2016 Adult Training and Education Survey to find that more than half of adults with a short-term certificate of up to 15-week duration who were employed earned $30,000 or less per year, which is below the national poverty line for a household of four. She found further that ‘the median yearly income for Black and Latino/Latina adults with a short-term certificate is $10,000 to $20,000 less than the median yearly income of their white counterparts who hold a credential of similar length.’ Some 41% of graduates with a short-term certificate were unemployed and of those who were employed, 39% reported that their current job was not related to their credential (Ositelu, 2021, pp. 14-15).

### Disciplining universities

Technologies like micro-credentials are said to be needed to disrupt universities because they are seen to be unresponsive, slow to move, and lacking motivation. For example, Chakroun and Keevy (2018 p.29) say that:

The fact that micro-credentials are more flexible and responsive make them very useful in designing flexible pathways that appeal to employers, compared with traditional or macro-credentials and their more static pathways which are often not well understood by employers.

This deficit language about universities and other higher education institutions is not new, particularly in their success or otherwise in embedding employability skills within curriculum (see Cotronei-Baird, 2020). This is precisely the language that has been used in the vocational education sector over the last 40 years to characterise vocational colleges (such as further education colleges in England or TAFE in Australia). They have been cast as inefficient and needing disciplining by the market, because they were said to teach outdated programs that employers didn’t want and students didn’t need, leading to skills mismatches, and declines in productivity because employers couldn’t get graduates with the ‘right’ skills (Bjørnåvold & Coles, 2007/8). The ‘solution’ in vocational education in countries such as Australia, the UK, and South Africa was to impose competency-based training tightly in which units of learning were aligned to workplace tasks and roles, leading to ‘tick and flick’ of the minutely specified learning outcomes in a teacher-proof curriculum (Allais, 2012).

This discipline is now being introduced into higher education, including universities. While the mechanisms for control in higher education are not as overt as they are for vocational education in most countries, nonetheless decades of neoliberal policies that have sought to subordinate and align all education to the labour market have affected universities. Funding is often a key mechanism to elicit compliance with a strong labour market focus. For example, in Australia in 2020, the Minister for Education, Dan Tehan (2020c) increased the fees that humanities students must pay by 113% because they putatively don’t result in students gaining good jobs. Students in the humanities n public universities will now pay more in fees than those studying under-graduate programs like medicine, dentistry, or veterinary science (Department of Education Skills and Employment, 2020, p.19). The Ontario government in Canada is introducing performance-based funding in which 60% of the government grant to colleges and universities will depend on performance against 10 metrics which focus on skill and job outcomes, and economic and community impact (Ministry of Colleges and Universities, 2020).

### Privatisation and marketisation

Micro-credentials facilitate the blurring of the public/private divide in higher education and contribute to the marketisation and creeping privatisation of public higher education through privatising the provision of micro-credentials. For example, FutureLearn, a prominent provider of micro-credentials, is jointly owned by the UK Open University (a public university) and Seek Ltd.^3^ FutureLearn boasts many partners including leading public universities, not-for-profit entities, and for-profit entities.^4^

Privatisation is occurring by enmeshing public universities in complex relationships with private-for-profit companies in delivering education. Public universities have long delegated ancillary services such as accommodation and extra curricula life to associated entities such as colleges. But many if not most are now contracting out to private providers the provision of core academic services such as library resources and learning management systems, and some are contracting out the provision of preparatory programs, language support, and supplementary tutoring (Wekullo, 2017). The stakes become profit, and driving down costs to drive up profit. This contributes to the commodification of learning and the ‘unbundling’ of curriculum in the service of greater efficiency and profit (Ralston, 2020). As an illustration, for $969, it is possible to undertake an online micro-credential in ‘Customer Experience Management with Salesforce Training’, which includes training in a company’s proprietary software, and receive 15 credits towards an MBA in Global Business at a university in London.^5^ Gone are the days where one could expect a company to train new recruits in their own proprietary software; in many cases, recruits must pay for this training themselves and come ready to start work on day one (Brown & Souto-Otero, 2018). Means (2018, p.326) explains that aspirations by the ‘Institute for the Future’ based in the Silicon Valley are that: ‘Every interaction, every gig, every book, every online module, and every micro-project is converted into edublocks, which are the basis of micro-credentials’ stored through blockchain technology.

The blurring of the distinction between public and private occurs by constituting a ‘market place’ of public and private providers which offer credentials increasingly interchangeably with each other, and this blurs distinctions between provider type. For example, in a foreword to Inside Higher Ed’s special report on *Alternative credentials and emerging pathways between education and work*, Lederman (2020, p.iii) explains:

The expansion of alternative credentials is helping to blur boundaries between credit-bearing and noncredit programs, between colleges and noncollege providers, and between higher education and post– high school job training. Defined broadly, alternative credentials and the competency-based learning systems that undergird them will change how many colleges operate, and warrant attention from faculty members and college leaders.

The distinction between those who work in public and private institutions is also blurring. In an illustrative example, Ralston (2020, p.3) reports that there is a ‘revolving door’ between senior executives in online divisions in universities and third party vendors of online platforms which underpin many micro-credentials.

## Conclusion

The key argument in this paper is that micro-credentials are gig credentials for the gig economy. Rather than presenting new opportunities for social inclusion and access to education, they contribute to the privatisation of education by unbundling the curriculum and blurring the line between public and private provision in higher education. Micro-credentials can contribute to the fragmentation of occupations by undermining the coherence of qualifications and occupations (Wheelahan, 2016). Micro-credentials contribute to ‘disciplining’ higher education in two ways: first by building tighter links between higher education and workplace requirements (rather than whole occupations), and,through ensuring universities are more ‘responsive’ to employer demands in a competitive market crowded with other types of providers.

Instead of micro-credentials, progressive, democratic societies should seek to ensure that all members of society have access to a substantial qualification that has value in the labour market and in society more broadly, and as a bridge to further education (Moodie et al., 2019; Wheelahan & Moodie, 2017). This is a broader vision of education in which the purpose of education is to prepare individuals to live lives they have reason to value (Nussbaum, 2000; Sen, 1999), and not just in the specifics required of particular jobs. Living a life that one has reason to value must have as part of its core, educational preparation for a career and meaningful work, underpinned by an agential worker who has a stake in and contributes to the evolution of their occupation (Winch, 2014), and who has a stake in and contributes to society more broadly (Bernstein, 2000).

## Footnotes

1 Iwi refers to a people or nation in Aotearoa (New Zealand) Māori society. There are different iwi in Aotearoa NZ. See: https://en.wikipedia.org/wiki/Iwi

2 See: https://worldreferencelevels.org/

3 See: https://www.futurelearn.com/info/blog/futurelearn-announces-seek-as-new-50-50-shareholders-alongside-the-open-university.

4 See https://www.futurelearn.com/partners. Partners include Microsoft, and for $694, students can do a micro-credential that includes Microsoft Azure, and undertake the Microsoft exam to achieve Microsoft certification. See: https://www.futurelearn.com/microcredentials/microsoft-ai-track

5 See: https://www.futurelearn.com/microcredentials/customer-experience-success-salesforce
